# Empfehlungen der Kommission Komplementäre Heilverfahren und Ernährung zu ayurvedischer Medizin, Homöopathie, Ernährung und mediterraner Kost

**DOI:** 10.1007/s00393-023-01356-z

**Published:** 2023-05-22

**Authors:** Gernot Keyßer, Andreas Michalsen, Monika Reuß-Borst, Inna Frohne, Mandy Gläß, Alexander Pfeil, Olaf Schultz, Olga Seifert, Oliver Sander

**Affiliations:** 1 Klinik für Innere Medizin II, Universitätsklinikum Halle (Saale), Ernst-Grube-Str. 40, 06120 Halle, Deutschland; 2 Immanuel Krankenhaus Berlin, Königstr. 63, 14109 Berlin-Wannsee, Deutschland; 3 Facharztpraxis für Innere Medizin, Frankenstr. 36, 97708 Bad Bocklet, Deutschland; 4 Privatpraxis für Rheumatologie, Frankenstr. 238, 45134 Essen, Deutschland; 5 Helios Fachklinik Vogelsang-Gommern, Sophie-von-Boetticher-Str. 1, 39245 Vogelsang-Gommern, Deutschland; 6 Klinik für Innere Medizin III, Universitätsklinikum Jena, Am Klinikum 1, 07747 Jena, Deutschland; 7 Rheumazentrum, ACURA Kliniken Baden-Baden, Rotenbachtalstr. 5, 76530 Baden-Baden, Deutschland; 8 Klinik und Poliklinik für Rheumatologie, Universitätsklinikum Leipzig, Liebigstr. 20, Haus 4, 04103 Leipzig, Deutschland; 9 Klinik für Rheumatologie, Universitätsklinikum Düsseldorf, Moorenstr. 5, 40225 Düsseldorf, Deutschland

**Keywords:** Komplementäre Medizin, Alternative Medizin, Rheumatologie, Rheumatische Erkrankungen, Praxisalltag, Complementary medicine, Alternative medicine, Rheumatology, Rheumatic diseases, Routine practice

## Abstract

Methoden der komplementären und alternativen Medizin („complementary and alternative medicine“ [CAM]) stoßen bei vielen Patienten mit rheumatischen Erkrankungen auf Interesse. Die wissenschaftliche Datenlage ist durch eine große Anzahl von Publikationen bei einem eklatanten Mangel an verwertbaren klinischen Studien gekennzeichnet. Anwendungen der CAM stehen im Spannungsfeld zwischen dem Bemühen um eine evidenzbasierte Medizin und um qualitativ hochwertige Therapiekonzepte auf der einen und wenig fundierten bis eindeutig unseriösen Angeboten auf der anderen Seite. Die Deutsche Gesellschaft für Rheumatologie (DGRh) hat 2021 eine Kommission Komplementäre Heilverfahren und Ernährung ins Leben gerufen, welche die aktuelle Evidenz für CAM-Anwendungen und ernährungsmedizinische Maßnahmen in der Rheumatologie sichten und in praktisch anwendbare Empfehlungen einarbeiten soll. Für die vorliegende Publikation wurden für 4 Bereiche Empfehlungen für den rheumatologischen Praxisalltag erstellt: Ernährung, mediterrane Kost, ayurvedische Medizin und Homöopathie.

## Abstract (en)

Methods of complementary and alternative medicine (CAM) are appealing for many patients with rheumatic diseases. The scientific data are currently characterized by a large number of publications that stand in contrast to a remarkable shortage of valid clinical studies. The applications of CAM procedures are situated in an area of conflict between efforts for an evidence-based medicine and high-quality therapeutic concepts on the one hand and ill-founded or even dubious offers on the other hand. In 2021 the German Society of Rheumatology (DGRh) launched a committee for CAM and nutrition, which aims to collect and to evaluate the current evidence for CAM applications and nutritional medical interventions in rheumatology, in order to elaborate recommendations for the clinical practice. The current article presents recommendations for nutritional interventions in the rheumatological routine for four areas: nutrition, Mediterranean diet, ayurvedic medicine and homeopathy.

## Einführung

Die Therapie rheumatischer Erkrankungen hat in den letzten Jahrzehnten eine Revolution erfahren. Studierende der Medizin kennen eine Reihe von charakteristischen Schadensbildern oft nur noch aus Lehrbüchern, weil diese weitgehend vermeidbar geworden und aus dem Alltag verschwunden sind. Die Erfolge der Treat-to-Target-Therapie beruhen häufig auf einer erstklassigen Evidenz, die Sicherheit der eingesetzten Medikamente hat ein hohes Niveau erreicht.

Interessanterweise hat diese erfreuliche Entwicklung nicht dazu geführt, dass das Interesse von ÄrztInnen und PatientInnen an Methoden der komplementären und alternativen Medizin („complementary and alternative medicine“ [CAM]) nachgelassen hätte. Unter den Stichworten „complementary medicine and arthritis“ finden sich in der PubMed-Datenbank aktuell (Stand 14.03.2023) über 5000 wissenschaftliche Artikel, die Hälfte davon wurde in den letzten 10 Jahren publiziert.

## Häufigkeit von CAM-Anwendungen in der Rheumatologie

CAM-Anwendungen sind weltweit unter Patienten mit entzündlich rheumatischen und degenerativen Gelenkerkrankungen stark verbreitet. Veröffentlichungen berichten, dass 24 % der Patienten mit Gichtarthritis [1], 40 % mit Spondyloarthritiden [2], 47 % mit Kniegelenkarthrose [3] und 75–95 % der PatientInnen mit entzündlichen Gelenkerkrankungen [4–6] jemals CAM angewendet haben, wobei deutlich weniger Patienten sich als aktuelle CAM-Anwender („current user“) bezeichneten. Zahlen aus Deutschland belegten, dass zwischen den Jahren 2000 und 2001 14 % der RA-Patienten einer Früh-RA-Kohorte CAM anwendeten [7]. Methoden der CAM werden oft miteinander und mit konventionellen Medikamenten kombiniert [8]. Führend in der CAM-Anwendung sind Nahrungsergänzungsmittel [1, 3, 4] bzw. Modifikationen der Ernährung [6]. Die CAM-Anwendung variiert zwischen ethnischen Gruppen [6, 8]. Weibliches Geschlecht und höherer Bildungsabschluss prädisponieren zur Anwendung von CAM [4] ebenso wie das Vorhandensein von gelenkbezogenen Symptomen als Zeichen einer aktiven Erkrankung [9].

Verfahren der CAM stellen in den USA fast 10 % der Gesundheitsausgaben von Patienten dar und bilden einen Markt von 28–30 Mrd. US-Dollar [10]. In Deutschland gaben nach Daten von 2004 PatientInnen mit früher RA im Durchschnitt 47 ± 250 € pro Jahr für alternative Medizin aus [7].

## Gründe für die Anwendung von CAM

Es gibt viele Erklärungen für die Motivation, auf CAM zurückzugreifen. Der Patientenwunsch, selbst die Kontrolle über die Erkrankung zu erlangen, zählt ebenso dazu wie Berichte aus Massenmedien und sozialen Netzwerken und die weit verbreitete Auffassung, dass die CAM-Anwendung risikofrei sei (Übersicht in [8]). Empfehlungen zur CAM-Anwendung erfolgen häufig durch Freunde und Familienmitglieder und nur selten durch Ärzte [11]. Eine proaktive Einstellung zur eigenen Erkrankung findet sich bei PatientInnen mit höherem Bildungsstand häufiger, daher korreliert die Intensität der CAM-Anwendung in einer Reihe von Erhebungen mit dem Bildungsstand der Betroffenen (Übersicht in [8]). Je nach Organisation des Gesundheitssystems im untersuchten Land werden auch Kostengründe für die Anwendung preiswerter CAM-Verfahren genannt [12]. Nicht selten ist auch eine allgemeine Skepsis vor der modernen Medizin und die Furcht vor noch unbeschriebenen Nebenwirkungen Anlass für die CAM-Anwendung [5, 12].

## CAM im Spannungsfeld zwischen „Schulmedizin“ und „Alternativmedizin“

Das Verhältnis zwischen Anwendern der „konventionellen“ oder „Schulmedizin“ und Verfechtern alternativmedizinischer Verfahren ist nicht unbelastet. Eine epidemiologische Studie zur frühen RA in Deutschland zeigte, dass Patienten, die alternativmedizinische Verfahren präferierten, hochsignifikant seltener eine antirheumatische Basistherapie einnahmen und somit dem Risiko einer Unterversorgung ausgesetzt waren [13]. Ein Verzicht auf moderne Medikamente zugunsten einer einseitigen Anwendung von CAM kann im Einzelfall erhebliche Nachteile für Patienten nach sich ziehen [14].

Eine Besonderheit der deutschen Medizin ist die Tatsache, dass Verfahren der CAM auch von HeilpraktikerInnen ausgeübt werden dürfen, ein Berufsstand, der in keinem anderen Mitgliedsstaat der EU existiert. Die gesetzliche Grundlage für dieses Gewerbe stammt aus dem Jahr 1939. Für die Ausübung dieses Berufes ist lediglich eine Überprüfung durch das Gesundheitsamt, jedoch keine Approbation erforderlich. Eine Ausbildung ist freiwillig und nicht staatlich geregelt, sie kann z. B. im Selbststudium oder online erfolgen und erfordert keine praktische Erfahrung [118]. Auf der anderen Seite können ÄrztInnen mit Facharztabschluss im Bereich der unmittelbaren Patientenversorgung die Zusatzbezeichnung „Naturheilverfahren“ erwerben, welche sie nach aktueller Weiterbildungsordnung befähigt, Verfahren der CAM anzuwenden (Tab. 1). In Deutschland besaßen 2021 nach Statistik der Bundesärztekammer über 16.400 ÄrztInnen diese Zusatzbezeichnung, die meisten von ihnen waren in der Allgemeinmedizin tätig. Der Berufsverband Deutscher Rheumatologen verfügt aktuell nicht über Daten, die angeben, wie viele RheumatologInnen diese Zusatzbezeichnung besitzen.

**Table 1:** 

Weiterbildungsinhalt	Beispiel
*Diagnostische Verfahren in der Naturheilkunde*	Manuelle Untersuchungen einschließlich Befunderstellung, z. B. am muskuloskeletalen Apparat
*Therapie mit Arzneimitteln und Nahrungsergänzungsmitteln*	Indikationsbezogene Therapie mit – Phytotherapeutika – Mikronährstoffen – Präbiotika und Probiotika
*Kneipp‑, Hydro‑, Balneo- und Klimatherapie*	Indikationsstellung und Beratung zu – Kneipp-Anwendungen – Hydrotherapie – Thermotherapie – Kryotherapie – Balneo- und Klimatherapie – Thalassotherapie
*Physikalische Verfahren*	Indikationsstellung und Beratung zu – Ultraschalltherapie – Foto- und Lichttherapie – Elektrotherapie einschließlich Magnetfeldtherapie
*Massagebehandlungen, Reflextherapie*	Indikationsstellung und Beratung zu – klassischer Massage – Bindegewebsmassage – Lymphdrainage – Kolonmassage – Periostmassage – Reflextherapie
*Manuelle Verfahren*	Indikationsstellung und Beratung zu – manuellen Verfahren – osteopathischen Verfahren
*Ernährung und Fasten*	Erkennung von Fehl- und Mangelernährung – Beratung zu – vollwertiger Ernährung – Fasten – Ernährungsänderungen bei entzündlichen, metabolischen und onkologischen Erkrankungen
*Ordnungstherapie, *Mind-Body-Medicine	Kenntnisse über den Einfluss psychosozialer Faktoren auf die Gesundheit
	Beratung zu Salutogenese, z. B. Lebensstil, Entspannung, Achtsamkeit
*Bewegungs- und Atemtherapie*	Indikationsstellung und Beratung zu Bewegungs- und Atemtherapieverfahren
*Ausleitende und umstimmende Verfahren*	Indikationsstellung und Durchführung von – Schröpfen – Blutegeltherapie – Eigenbluttherapie – Aderlasstherapie
	Indikationsstellung und Beratung zu diuretischen und laxierenden Verfahren
*Grundlagen der Neuraltherapie und Akupunktur*	Indikationsstellung und Durchführung von Neuraltherapie, davon – Quaddelbehandlungen – Segmentinfiltration – Narbeninfiltration

Eine vorurteilsfreie Diskussion über die Charakteristika, Potenziale und Limitationen beider Therapieprinzipien ist dazu geeignet, Verunsicherungen bei Patienten abzubauen und das Vertrauensverhältnis zu ihren ÄrztInnen zu stärken. Nur etwa die Hälfte der Patienten, die CAM anwenden, berichtet darüber ihren behandelnden Ärzten [8]. Eine offene, partizipative Kommunikation mit den Patienten führt nachweislich dazu, dass diese unbefangener über ihre Erfahrungen mit CAM berichten [15]. Bei der Bewertung der Effektivität von CAM dürfen Prinzipien der evidenzbasierten Medizin nicht missachtet werden. Allerdings ist dabei zu berücksichtigen, dass therapeutische Ansätze von CAM oft auf Bereiche zielen, die in zentralen Outcome-Parametern wie dem DAS28 nur mittelbar abgebildet werden. Stattdessen zielen sie häufig auf Kriterien der Patientenperspektive, die sog. „patient reported outcomes“ (PRO). Dazu gehören die mentale Leistungsfähigkeit, körperliche Fitness, emotionale Stabilität oder Schlafqualität [16].

Eine offene Kommunikation über CAM kann auch verhindern, dass PatientInnen durch alternativmedizinische Methoden geschädigt werden. Nicht alle Verfahren der CAM sind unbedenklich. Nahrungsergänzungsmittel können über Unverträglichkeitsreaktionen zu Notfallsituationen führen [17], hoch dosierte Vitaminpräparate zu einer Übersterblichkeit beitragen [18] und extreme Diätformen Mangelerscheinungen auslösen [19]. Nebenwirkungen und Interaktionen pflanzlicher Präparate mit anderen Pharmaka sind v. a. bei älteren Patienten möglich [20].

## CAM und evidenzbasierte Medizin

Wissenschaftlich begründbare Aussagen über die klinische Wirksamkeit von CAM zu treffen ist anspruchsvoll. Die Wirkmechanismen von Methoden der CAM sind oft nur in Ansätzen untersucht. Die theoretischen Grundlagen einiger CAM-Verfahren wie die Homöopathie halten einer wissenschaftlichen Nachprüfung nicht stand [21]. Eine Reihe von antientzündlichen Mechanismen von Phytotherapeutika ist bekannt, diese stammen aber in der Mehrzahl aus In-vitro-Daten (Übersicht in [22]). Klinische CAM-Studien sind oft schlecht zu verblinden, publizierte Fallzahlen sind in der Regel klein und nicht oder schlecht kontrolliert, die Placeboeffekte können ausgeprägt sein [10, 23]. Komplexe therapeutische Systeme wie die ayurvedische Medizin oder die traditionelle chinesische Medizin beruhen auf individuellen, führend langfristig präventiven Interventionen und lassen sich daher in der Matrix einer klar umrissenen klinischen Studie schlecht bzw. nur mit großem Aufwand definieren und vereinheitlichen [24]. Diese Defizite können durch Fortschritte bei der Analyse und Nachbereitung von Literaturdaten durch Methoden der künstlichen Intelligenz, z. B. durch bibliometrische Analysen [25] oder „data mining“ [26], nur ansatzweise kompensiert werden.

Für die RA wird die vorliegende Evidenz für die Wirksamkeit von Verfahren der CAM als schwach bewertet [10, 27] und auf mögliche Nebenwirkungen einiger Phytotherapeutika hingewiesen [27]. Eine mäßige Evidenz für eine Wirksamkeit bei RA wird lediglich einigen Methoden der Mind-Body-Medizin sowie der mediterranen Kost, dem therapeutischen Fasten und dem Einsatz von Omega-3-Fettsäuren zugestanden [10]. Für die Spondyloarthritiden werden physikalische Verfahren wie Tai Chi positiv bewertet, Eingriffen in das Mikrobiom z. B. durch Probiotika wird zumindest das Potenzial eines therapeutischen Ansatzes attestiert [23]. Patienten mit Fibromyalgie könnten von der topischen Anwendung von Capsaicin oder von Akupunktur profitieren, aber auch hier ist die Evidenzlage unbefriedigend [28].

Für zahlreiche andere Erkrankungen des rheumatischen Formenkreises ist die Datenlage so dürftig, dass überhaupt keine Aussagen zur Wirksamkeit einzelner Komponenten der CAM getroffen werden können. Das betrifft v. a. die selteneren autoimmunen Systemerkrankungen. Daher erwähnen im internationalen Maßstab nur wenige Leitlinien zur RA oder Arthrose die Anwendung von CAM. Empfehlungen werden, wenn überhaupt, meist zu etablierten physiotherapeutischen Verfahren wie manueller, Elektro- oder Thermotherapie ausgesprochen [29].

## Empfehlungen der Kommission Komplementäre Heilverfahren und Ernährung

Die Kommission Komplementäre Heilverfahren und Ernährung wurde am 06.04.2021 auf Anregung des Vorstandes der DGRh gegründet. Ihr Ziel ist es, einzelne Verfahren der CAM im Hinblick auf ihre wissenschaftliche Datenlage zu untersuchen und Empfehlungen für oder gegen ihre Anwendbarkeit in der Rheumatologie zu erarbeiten. Die Mitglieder der Kommission haben sich nach anfänglicher Sichtung möglicher Themenfelder der CAM für die im Titel genannten 4 Bereiche entschieden. Diese wurden zunächst von einzelnen Mitgliedern der Kommission nach folgenden Gesichtspunkten bearbeitet:

- Definition der Methode im Sinne einer in der Praxis verständlichen und nachvollziehbaren Darstellung;
- Überblick über die wissenschaftliche Evidenz in der Literatur. Dazu nahmen die Kommissionsmitglieder Recherchen in medizinischen Datenbanken vor – in erster Linie in der PubMed-Datenbank der National Library of Medicine (USA), aber auch MEDLINE und PubMed Central (PMC);
- mögliche Anwendungen in der Rheumatologie inklusive zu erwartender positiver Effekte;
- mögliche Nebenwirkungen und Limitationen.

Für die beiden letztgenannten Punkte wurden ebenfalls Recherchen in den genannten Datenbanken vorgenommen. In 2 Bearbeitungsrunden wurden die erarbeiteten Entwürfe anschließend in der Kommission besprochen, und eine gemeinsame Empfehlung wurde erstellt. Diese wurde dem Vorstand der DGRh zugeleitet und nach deren abschließenden Kommentaren in der hier vorliegenden Form verabschiedet.

## Empfehlungen der Kommission für Komplementäre Heilverfahren und Ernährung zur Modifikation der Ernährung als supportive Maßnahme bei rheumatischen Erkrankungen

### Definition der Methode

Aus eigener Erfahrung ist Patienten und Ärzten bekannt, dass die Ernährung einen Einfluss auf Krankheitsaktivität und Schubfrequenz entzündlich rheumatischer Erkrankungen haben kann [30, 31]. In den letzten Jahren rückte deshalb der Stellenwert einer geeigneten Ernährungsform in den Fokus des wissenschaftlichen Interesses. So gibt es Hinweise für den Einfluss der Ernährung oder einzelner Makro- und Mikronährstoffe auf eine Vielzahl von Pathomechanismen der rheumatoiden Arthritis (RA) und weiterer entzündlich rheumatischer Erkrankungen in experimentellen Modellen. Im Vordergrund stehen dabei: 1) proinflammatorische, antiinflammatorische oder antioxidative Effekte einzelner Nährstoffe oder ganzer Ernährungsformen, 2) Effekte auf die Darmmikrobiota und das Darm-assoziierte Immunsystem, 3) Effekte von Ernährung auf wesentliche Komorbiditäten rheumatischer Erkrankungen (Diabetes mellitus, Herz-Kreislauf-Erkrankungen), 4) Gewichtsnormalisierung bei relevanter Adipositas.

Die Zahl klinischer kontrollierter und randomisierter Studien zur Ernährungsmodifikation bei rheumatischen Erkrankungen ist allerdings noch immer überschaubar, was u. a. auf die Schwierigkeiten beim Studiendesign und der Finanzierung zurückzuführen ist. So stammen die meisten Empfehlungen aus Beobachtungsstudien, in denen bestimmte Ernährungsmuster unter nicht-kontrollierten Bedingungen untersucht wurden. In diesem Kapitel wird die derzeitige Datenlage vorgestellt, wobei nicht auf einzelne Nahrungsbestandteile, sondern die aktuell bekannten Effekte bestimmter Ernährungsformen, wie z. B. mediterrane Ernährung (s. dazu auch eigenes Kapitel der Kommission), eingegangen wird. Insgesamt ist die klinische Evidenz für den rheumatischen Formenkreis deutlich schwächer im Vergleich zu kardiovaskulären und metabolischen Erkrankungen und bezieht sich mit wenigen Ausnahmen nur auf die RA.

### Überblick über wissenschaftliche Evidenz in der Literatur

Die Arbeitsgruppe von Kjelsen-Kragh aus Oslo hatte in den 90er-Jahren umfangreiche klinisch-experimentelle Arbeiten zum Nutzen von Fastenperioden und/oder vegetarischen Diäten durchgeführt. In einer darauf aufbauenden randomisiert kontrollierten Studie zeigte sich ein therapeutischer Nutzen einer initialen Fastentherapie, gefolgt von pflanzlicher und glutenfreier Nahrung und nachfolgend (aufbauend) lactovegetarischer Ernährung über die Studiendauer von 13 Monaten [32].

In einer weiteren randomisierten Studie zeigte sich eine klinische Besserung unter einer veganen und glutenfreien Ernährung, verglichen mit einer vollwertigen Normalkost [33]. Der Verum-Arm wies allerdings eine hohe Abbruchrate auf.

Sköldstam et al. publizierten die erste randomisierte Studie zur mediterranen Diät bei RA. Es zeigte sich nach 6 und 12 Wochen der Aufnahme einer mediterranen Diät der DAS28 in der Interventionsgruppe bei 15/26, in der Kontrollgruppe bei 6/23 Patienten um mindestens 0,6 Punkte verbessert, dabei bestand kein Unterschied zwischen den Gruppen bezüglich einer Verbesserung um mindestens 1,2 Punkte (EULAR Response) [34]. In einer größeren, kontrollierten, aber nicht randomisierten Studie zur Umsetzung mediterraner Kost bei Patienten mit rheumatoider Arthritis in einer sozial benachteiligten Population wurde gezeigt, dass regelmäßige Schulungs- und Kochkurse nötig sind, um überhaupt eine Verhaltensänderung in dem Ausmaß zu erreichen, dass Besserungen von patientenzentrierten Outcomes wie HAQ oder Morgensteifigkeit in einem Beobachtungszeitraum von 6 Monaten erreicht werden [35] (s. auch Abschn. „Empfehlungen der Kommission für Komplementäre Heilverfahren und Ernährung zur mediterranen Ernährung“).

Eine unlängst publizierte randomisierte Cross-over-Studie (ADIRA Trial) untersuchte die Effekte einer vollwertigen Ernährung mit Schwerpunkt auf Gemüse, Obst, Fisch, reduziertem Fleischverzehr und Probiotika als Nahrungsergänzung. Von 50 Teilnehmern konnten 47 ausgewertet werden. Nur in der unadjustierten Analyse ergab sich ein Vorteil für die Intervention nach 10 Wochen im DAS28. Die Studie erscheint jedoch „underpowered“ und zu kurzzeitig in der jeweiligen Beobachtungsphase [36]. Neue Konzepte umfassender antirheumatischer Diäten finden sich in der klinischen Evaluation [37].

### Reviews

Eine Cochrane-Analyse der skandinavischen Arbeitsgruppe um Hagen identifizierte 2009 14 RCTs („randomized controlled trials“) und eine kontrollierte Studie mit insgesamt 837 Patienten. Die Autoren zogen den Schluss: „Die Effekte von diätetischen Eingriffen, einschließlich vegetarischer und mediterraner Diät, Elementardiät und Eliminationsdiäten auf die rheumatoide Arthritis sind noch immer nicht belastbar gesichert, da es sich bei den eingeschlossenen Studien um kleinere Einzelstudien mit mäßigem bis hohem Risiko für einen Bias handelt.“ (The effects of dietary manipulation, including vegetarian, Mediterranean, elemental and elimination diets, on rheumatoid arthritis are still uncertain due to the included studies being small, single trials with moderate to high risk of bias) [19].

Ein aktueller systematischer Review zu RCTs bei RA identifizierte 27 Publikationen zu Ernährungsinterventionen und ihren Effekten auf die Krankheitsaktivität, gemessen mit dem DAS28. Herausgehoben wurden 3 Studien (mediterrane Diät, vegane Kost, antiinflammatorische Diät). Mögliche positive Effekte für einzelne hoch dosierte Gewürze wie Ingwer, Zimt und Safran wurden betont. Die Autoren folgerten, dass es nur wenige aussagekräftige Studien gibt, aber die Vermutung besteht, dass einige Interventionen, wie z. B. Antioxidanzien wie das Polyphenol Quercetin oder *Lactobacillus casei* enthaltende Probiotika, positive Effekte auf die Krankheitsaktivität der RA, gemessen mit dem DAS28, haben könnten [38].

Ein weiterer, aktueller Review identifizierte 70 Studien zu den Themen Ernährung, Fasten und Nahrungsergänzungen. Die Autoren fanden eine mögliche günstige Wirkung von Vitamin-D-Supplementation und Salzrestriktion für einzelne RA-Outcomes. Fasten wurde als kurz- bis mittelfristig wirksam eingeordnet. Während für die mediterrane Ernährung eine klinische Besserung als mäßig belegt beschrieben wurde, werden die Ergebnisse für vegetarische und Eliminationsdiäten nicht als konsistent bewertet, und ein individuell schwankendes Ansprechen wird vermutet [39].

In einem aktuellen systematischen Review von 20 RCTs mit 717 RA-Patienten wird die Ergänzung von Omega-3-Fettsäuren als günstig auf klinische Outcome-Parameter bewertet [40]. Eine sehr viel umfassendere Datenlage findet sich in der Kardiologie. Hier haben die großen Studien den Nutzen von einer Supplementierung von Omega-3-Fettsäuren nicht bestätigt. Lediglich für einen speziellen Eicosapentaensäure-Ester konnte die Wirksamkeit belegt werden [41]. Eine Empfehlung zur Supplementierung mit Omega-3-Fettsäuren kann für die RA nicht gegeben werden.

### Fasten

In einer bereits 1991 publizierten Studie von Kjeldsen-Kragh [32] wurden die günstigen Wirkungen einer Fastenkur von 7 bis 10 Tagen dokumentiert. Diese Ergebnisse wurden allerdings vor der Ära der modernen medikamentösen Therapie der RA erzielt. Ebenso stammt die dargelegte metaanalytische Evidenz von 4 Studien von Fasten bei RA [42] noch aus der Präbiologikaära. Empirisch gibt es eine langjährige Erfahrung zu den beschwerdelindernden und entzündungshemmenden Wirkungen des Fastens in spezialisierten Kliniken. Hierbei umfassen diese Daten aus Registern und aus den erwähnten Studien (mit Ausnahme von Kjeldsen-Kragh [32]) nur kurzzeitige Zeiträume von 6 bis 12 Wochen. Daten zu langfristigen Effekten wiederholter Fastenregimes fehlen genauso wie Daten zum Einfluss des Fastens auf den röntgenologischen Progress.

### Ketogene Ernährung

Einen Fasten-imitierenden Therapieansatz stellt die sog. ketogene Ernährungsform dar, bei der analog zum Fasten der Körper seine Energie überwiegend durch Verstoffwechselung von Ketonkörpern gewinnt [43]. Diese entstehen jedoch nicht wie beim Fasten durch Abbau von körpereigenem Fett, sondern durch Zufuhr von exogenen (idealerweise überwiegend pflanzlichen) Fetten. Hierzu ernähren sich die Patienten sehr fettreich und extrem kohlenhydratarm. Den dabei entstehenden Ketonkörpern (v. a. β‑Hydroxybutyrat) werden antioxidative und antiinflammatorische Effekte zugeschrieben [44, 45]. Auch wenn es sich um einen interessanten (Fasten-imitierenden) Therapieansatz handelt, gibt es bislang im Gegensatz zu anderen Indikationsgebieten wie Diabetes mellitus Typ 2 [46] keine klinischen Studien zu rheumatischen Erkrankungen [47].

### Mikrobiom

Eine Rolle des Mikrobioms wird bei der Entstehung rheumatischer Erkrankungen sowie zahlreicher anderer Autoimmunerkrankungen als sehr wahrscheinlich angesehen. Bei der New-Onset-RA sind distinkte Veränderungen der Mikrobiota dokumentiert [48].

Der wichtigste Einflussfaktor auf das Mikrobiom ist die Ernährung. Allerdings ist derzeit noch unklar, wie durch Ernährungsmaßnahmen gesundheitsfördernde Modifikationen im Mikrobiom konkret steuerbar sind. Hier sind die Forschungen der kommenden Jahre abzuwarten. Erste klinische Studien weisen darauf hin, dass ballaststoffreiche und fermentierte Nahrungsmittel durch Modifikation des Mikrobioms Entzündungsparameter reduzieren können und immunmodulierende Effekte entfalten [49, 50].

### Mögliche Anwendungen in der Rheumatologie inklusive zu erwartender positiver Effekte

Anhand von Tiermodellen [51–56] und aufgrund der vorliegenden klinischen Studien besteht weitgehender Konsens über folgende Einordnungen der Makronährstoffe Fett und Kohlenhydrate:

#### Fette.

Arachidonsäure und ihre Präkursoren wirken in vitro und im Tiermodell proinflammatorisch und sollten reduziert werden, das gilt ebenso für gesättigte Fette und einzelne Metabolite wie Trimethyl-Aminoxyd (TMAO), ein Abbauprodukt aus dem Phospholipid Cholin. Diese Fette finden sich jeweils in Fleisch/Wurst, entsprechend ist ein reduzierter Fleischverzehr als günstig ableitbar.

Omega-3-Fettsäuren (O-3-FS) pflanzlicher oder mariner Herkunft sind im experimentellen Modell antiinflammatorisch wirksam. Ein Ersatz gesättigter Fettsäuren durch Verzehr von O‑3-FS enthaltenden Ölen, Gemüsen, Nüssen, Algen und Fisch erscheint im Rahmen einer gesundheitsorientierten Ernährung empfehlenswert. Konkrete Dosisempfehlungen existieren nicht, zumal die Resorption der Omega-3-Fettsäuren interindividuell stark unterschiedlich ist. Außerdem kann ihre Bioverfügbarkeit z. B. durch eine fettreiche Mahlzeit gesteigert werden [57].

#### Kohlenhydrate.

Für raffinierte Zucker sind ungünstige kardiovaskuläre, adipogene sowie leicht proinflammatorische Wirkungen belegt [58]. Spezifische Studiendaten bei etablierten rheumatischen Erkrankungen fehlen aber. Für Gluten werden experimentell proinflammatorische Wirkungen auch ohne Bestehen einer Zöliakie beschrieben, allerdings fehlen konsistente Studiendaten für den Nutzen einer glutenfreien Diät.

### Mögliche Nebenwirkungen und Limitationen

Bei der Durchführung der hier diskutierten Ernährungsformen sind kaum Nebenwirkungen zu erwarten. Beim Fasten sollte auf eine ausreichende Flüssigkeitszufuhr geachtet werden, um z. B. das Auftreten von Gichtanfällen zu vermeiden. Im akuten Schub einer rheumatischen Erkrankung und bei schlecht kontrolliertem rheumatologischem Krankheitsbild soll nicht gefastet werden, ebenso nicht bei manifester Gicht und bei symptomatischen Gallensteinen. Bei Untergewicht und Essstörungen ist Fasten kontraindiziert. Gegebenenfalls kann es beim Fasten wie auch bei der ketogenen Ernährung in den ersten Tagen zu leichten Kopfschmerzen und anderen Befindlichkeitsstörungen kommen. Eine ketogene Ernährung ist zudem bei Typ-1-Diabetes sowie seltenen Fettoxidationsstörungen kontraindiziert. Generell sollten Ernährungsinterventionen, wie z. B. längere Fastenphasen, nur in Begleitung von geschultem Personal erfolgen.

### Abschließende Empfehlung der Kommission

Aufgrund der bislang vorliegenden Studien kann eine vollwertige, gemüse- und obstreiche (ballaststoffreiche) sowie fleischarme Ernährung Patienten mit entzündlich rheumatischen Erkrankungen als supportive, die Selbstwirksamkeit fördernde Maßnahme empfohlen werden. Dies gilt insbesondere auch mit Blick auf in Studien nachgewiesene gesundheitsförderliche Effekte einer solchen Ernährungsform auf häufig assoziierte Komorbiditäten (z. B. kardiovaskuläre Erkrankungen).

Die beste Evidenz dafür gibt es für die mediterrane Kost (s. auch Abschn. „Empfehlungen der Kommission für Komplementäre Heilverfahren und Ernährung zur mediterranen Ernährung“). Dabei sollte darauf hingewiesen werden, dass die klassische (insbesondere in Studien wie PREDIMED untersuchte) mediterrane Ernährung stets auch Kohlenhydrat-reduziert war, was v. a. den Anteil von raffiniertem Zucker anbelangt [59].

Die Kommission empfiehlt grundsätzlich, PatientInnen qualifiziert und bestmöglich über eine Ernährung zu informieren, die Entzündungsvorgänge hemmt oder zumindest nicht weiter verstärkt. Dafür sollten die Kapazitäten in den kommenden Jahren verbessert werden. Betont werden sollte dabei immer der supportive Therapieansatz – die medikamentöse Therapie sollte in jedem Fall fortgeführt werden. Wünschenswert wären zudem Langzeitstudien, insbesondere zu Adhärenz, Krankheitsverlauf und Langzeitprognose unter bestimmten Ernährungsformen. Künftige Forschungsarbeiten sollten sich auch mit der Frage beschäftigen, welche Phase des Krankheitsverlaufs den optimalen Startpunkt für ernährungsmedizinische Interventionen darstellt und in welcher Größenordnung die Krankheitsaktivität durch Ernährung überhaupt beeinflusst werden kann.

Eine routinemäßige Mikrobiomanalyse als Basis einer individualisierten Ernährungstherapie kann derzeit nicht empfohlen werden, da sich daraus keine anderen therapeutischen Empfehlungen als die derzeit geltenden ableiten lassen. Die Untersuchungen sind zudem kostenintensiv und werden nicht von den Krankenkassen übernommen.

Fastentherapien als klassisches Heilfasten über 5 bis 10 Tage oder in Form moderner „fasting-mimicking diets“ können bei fehlenden Kontraindikationen durch methodisch erfahrene ÄrztInnen überwacht und ergänzend zur Ernährungstherapie probatorisch eingesetzt werden. Da inzwischen eine wachsende Evidenz auf günstige Effekte des Fastens bei Bluthochdruck und Diabetes Typ 2 weist, kann Fasten zudem bei Vorliegen dieser Komorbiditäten empfohlen werden, insbesondere dann, wenn PatientInnen für eine solche Maßnahme motiviert sind.

## Empfehlungen der Kommission für Komplementäre Heilverfahren und Ernährung zur mediterranen Ernährung

### 1. Definition der Methode

Der Terminus „mediterrane Ernährung“ (ME) beschreibt traditionelle Ernährungsgewohnheiten des Mittelmeerraumes. Diese weisen abhängig von Ländern und Regionen eine hohe Variabilität auf, haben jedoch wichtige Elemente gemeinsam [60]. Dazu gehören

1. ein hoher Anteil pflanzenbasierter Nahrung: Früchte, Gemüse, Hülsenfrüchte, Nüsse, Brot, Zerealien,
2. Olivenöl als wichtigste Quelle von Fetten und ein niedriger Anteil gesättigter tierischer Fette (z. B. Butter und Schmalz),
3. Milchprodukte in Form von Käse, Joghurt und Kefir,
4. Fisch und Geflügel in variablen, aber im Vergleich zu durchschnittlicher „westlicher“ Ernährung geringen Anteilen bei einem deutlich reduzierten Anteil von „rotem“ Fleisch,
5. ein deutlich reduzierter Anteil von Weißzucker und Glukose-Fruktose-Sirup (enthalten z. B. in Softdrinks),
6. fakultativ: Weingenuss in geringen Mengen (nicht mehr als 10 g Alkohol pro Tag) zu den Mahlzeiten.

Diese Ernährungsform ermöglicht Vielfalt, kann sehr schmackhaft sein und ist wissenschaftlich gut untersucht.

### 2. Überblick über die wissenschaftliche Evidenz in der Literatur

Die Adhärenz zur ME kann in Scoresystemen quantifiziert werden. Dazu gehört der Alternate Mediterranean Diet Score (AMDS). Dieser wichtet die Anteile positiv bewerteter Nahrungskomponenten wie Gemüse, Hülsenfrüchte, Nüsse mit positiven Zahlen, negativ bewertete (z. B. rotes Fleisch) mit negativen Zahlen [61]. Ein hoher Scorewert ist in Querschnittstudien mit signifikant besserer Überlebenswahrscheinlichkeit assoziiert [61], zudem mit verringerter Wahrscheinlichkeit von Herzinfarkt und Apoplex [62]. Eine hohe Adhärenz zur ME geht mit geringeren Inzidenzen metabolischer Erkrankungen und Adipositas einher (Übersicht in [63]). In der longitudinalen Nurses Health Study korrelierte die Umstellung einer durchschnittlichen „westlichen“ Ernährung in Richtung eines höheren AMDS mit einer Verbesserung von Gesamtmortalität und Tod an kardiovaskulären Ursachen [64]. Eine große prospektive Studie zur Primärprävention von kardiovaskulären Ereignissen belegte einen protektiven Effekt der ME v. a. bei Anreicherung des Kostplans mit Olivenöl und Nüssen [59]. Eine ME lässt auch das Malignomrisiko sinken, v. a. im Hinblick auf kolorektale Karzinome [65]. Hier haben Vollkornprodukte, Gemüse und Früchte den größten Einfluss, ebenso ein geringer Alkoholkonsum zu den Mahlzeiten [65].

### 3. Wissenschaftliche Evidenz in der Rheumatologie

Wissenschaftliche Veröffentlichungen zum Einfluss der ME auf Erkrankungsrisiko und Verlauf finden sich nur bei wenigen rheumatischen Erkrankungen, die hier zusammengefasst sind:

#### Rheumatoide Arthritis (RA).

Der AMDS war in einer schwedischen Kohortenstudie invers korreliert mit dem Risiko für eine seropositive RA [66]. Hohe Adhärenz zur ME senkte das Risiko für eine RA verglichen mit niedriger Adhärenz um 21 %, der Effekt war allerdings nur bei Männern statistisch signifikant [66]. Auch in der Nurses Health Study war bei über 170.000 Frauen keine Korrelation des AMDS mit dem RA-Risiko nachweisbar [67].

Bei etablierter RA führte eine ME im Rahmen einer kontrollierten Interventionsstudie zu einer geringfügigen, aber signifikanten Verbesserung der entzündlichen Aktivität, gemessen am DAS28 und an Patient Reported Outcomes (PRO), darunter dem Health Assessment Questionnaire und der visuellen Analogskala für Schmerz [68].

In einer weiteren Studie erhielten RA-Patienten eine genau definierte antientzündliche Diät, die sich explizit auf die Prinzipien der ME stützte (ADIRA[Anti-Inflammatory Diet in RA]-Studie). Auch hier ließen sich im Verlauf der Intervention Verbesserungen des DAS28 und von einigen PROs nachweisen, auch wenn die Veränderungen diskret waren und im Vergleich zu einer Kontrollgruppe das Signifikanzniveau knapp verfehlten [36, 69]. Die Diät führte zu einer hochsignifikanten Verbesserung von Parametern des Fettstoffwechsels im Sinne einer weniger atherogenen Lipidkonstellation [70].

#### Psoriasisarthritis (PsA).

Für einen möglichen Zusammenhang zwischen ME und PsA-Inzidenz gibt es nur indirekte Hinweise: Bei Psoriasis vulgaris ist die Schwere der Hauterscheinungen [71], bei manifester PsA die arthritische Aktivität [72] invers mit der Adhärenz an die Prinzipien der ME korreliert.

#### Spondyloarthritiden (SpA).

Eine Schulung von SpA-Patienten zur Anwendung der ME führte zu leichtgradigen Verbesserungen der Krankheitsaktivität, gemessen am ASDAS(Ankylosing Spondylitis Disease Activity Score mit C-reaktivem Protein)-CRP [73].

#### Systemischer Lupus erythemathodes (SLE).

Das Erkrankungsrisiko für einen SLE wird offenbar nicht von der Ernährungsqualität im Allgemeinen und der Adhärenz zur ME beeinflusst [74]. Bei etabliertem SLE ging die Adhärenz zur ME in einer Querschnittstudie mit geringeren kardiovaskulären Risikofaktoren, einer geringeren SLE-Aktivität im SLEDAI (Systemic Lupus Disease Activity Index) und weniger krankheitsbedingtem Schaden einher [75].

### 4. Mögliche Anwendungen in der Rheumatologie inklusive zu erwartender positiver Effekte

Patienten mit den oben genannten Krankheitsbildern kann eine ME aufgrund der beschriebenen positiven Effekte auf die Erkrankungsaktivität als flankierende Maßnahme zur antirheumatischen Basistherapie empfohlen werden. Zudem kann die ME bei allen, auch den hier nicht aufgeführten rheumatischen Erkrankungen aufgrund der positiven Effekte auf das kardiovaskuläre Risikoprofil und die Inzidenz metabolischer Begleiterkrankungen empfohlen werden.

### 5. Mögliche Nebenwirkungen und Limitationen

Bei sachgemäßer Anwendung sind keine negativen Effekte zu erwarten. Die ME ist nicht gleichzusetzen mit einer veganen Kost, sodass eine zusätzliche Supplementierung z. B. mit Vitamin B_12_ nicht erforderlich ist. Das Risiko, dass Patienten mit starker Affinität zur komplementären Medizin eine notwendige medikamentöse Therapie durch eine Ernährungsumstellung „ersetzen“ möchten, ist zwar gering, sollte aber berücksichtigt werden.

### 6. Abschließende Empfehlung der Kommission

Die Kommission empfiehlt, dass RheumatologInnen grundsätzlich allen PatientInnen, die wegen entzündlich rheumatischer Erkrankungen in dauerhafter Betreuung sind, eine ME nahelegen und ihnen entsprechendes Informationsmaterial aushändigen. Dies gilt v. a. für Patienten mit zusätzlichen Risikofaktoren für kardiovaskuläre Erkrankungen. Schulungsangebote von Patientenselbsthilfegruppen oder die Möglichkeit der Überweisung zu einer professionellen Ernährungsberatung für die ME sollten genutzt werden. Eine derartige Intervention ist nicht zwingend erforderlich, wenn der Patient bereits eine weitgehend pflanzenbasierte Kostform einhält, die den Anforderungen an eine gesundheitsförderliche Ernährung entspricht.

## Empfehlungen der Kommission für Komplementäre Heilverfahren und Ernährung zur ayurvedischen Medizin bei rheumatischen Erkrankungen

### 1. Definition der Methode

Die Ursprünge des Ayurveda werden im Zeitraum von 1500 bis 800 vor Christus gesehen.

Die zentralen Konzepte der traditionellen indischen Medizin basieren auf systemeigenen Paradigmen mit zahlreichen Bezügen zu naturphilosophischen und erkenntnistheoretischen Systemen Südasiens. Vegetative/körperliche und geistige Funktionen sind verknüpft ähnlich der hiesigen psychosomatischen Lehre.

Körper und Geist sind die beiden ergänzenden Bestandteile eines Lebens, individuelle Homöostase ist verantwortlich für die Gesundheit und ihr Ungleichgewicht für die Entstehung von Krankheit. Ayurveda versucht, diese Kräfte in Balance zu halten, ist daher v. a. als Gesundheitsfürsorge und Prävention neben der Krankheitsbehandlung konzipiert [76].

In Indien gibt es seit 2015 ein eigenes Bundesministerium für traditionelle Medizinsysteme (AYUSH) sowie zahlreiche akademische Lehrinstitute und eigene Studiengänge für Ayurveda mit den Graden Bachelor of Ayurveda Medicine and Surgery Degree Course (B.A.M.S.), Doctor of Medicine in Ayurveda M.D. (Ayu), Bachelor und Master of Pharmacy in Ayurveda B.Pharm (Ayu). Laut AYUSH gibt es aktuell über 400.000 approbierte Ayurveda-Ärzte in Indien.

Der Diagnose und Behandlung geht in Ayurveda eine umfangreiche klinische Untersuchung und stark individualisierte Anamnese voraus. Der Patient wird nicht nur als erkranktes Wesen untersucht, sondern auch als Individuum mit den unten genannten Attributen einschließlich seiner Konstitution, seines Lebensstils und der jeweiligen Lebensumstände sowie möglicher externer Einflussfaktoren.

Einzeln bewertet werden: psychosomatische Konstitution; Krankheitsanfälligkeit; Qualität der Gewebe; Körperbau; Anthropometrie; Anpassungsfähigkeit; psychische Gesundheit; Ernährung und Verdauung; Ausdauer/Trainingszustand; Alter.

Die Untersuchung des Patienten umfasst u. a.: Puls, Zunge, Stimme und Sprache, Haut, Augen, Gesamterscheinung sowie Inspektion von Urin und Stuhl.

Als ein Grundanschauungsmodell verwendet Ayurveda das Konzept der 3 eigenschaftsbasierten Funktionsprinzipien *Vata* (kinetisch-kataboles Prinzip), *Pitta* (metabolisch-thermisches Prinzip) und *Kapha* (anaboles Prinzip), deren dynamische Interaktion im Organismus biologisches Leben definiert. Das Konzept dient auch zur Beschreibung gesundheitlicher Störungen. Therapeutisches Ziel ist die Wiederherstellung oder bestmögliche Annäherung an die individuelle Homöostase von *Vata, Pitta* und *Kapha*.

Die Therapien liegen v. a. in der gesundheitlichen Primärversorgung und umfassen mehrere Komponenten:

Nachfolgend werden einige Beispiele genannt:

*Rasayana* (Stärkungs- und Kräftigungstherapie) dient der Erhaltung und Förderung von Gesundheit. *Rasayana*-Therapien sollen Langlebigkeit fördern und Alterungsprozesse verzögern sowie Immunität und Resilienz begünstigen.

Ein weiteres Behandlungsmodul ist *Panchakarma* (stationäre Reinigungstherapie). Sie soll die Entfernung von Gift- und Abfallstoffen sowie Ablagerungen aus dem Körper bewirken, aber auch Krankheiten vorbeugen und Gesundheit fördern. *Panchakarma*-Verfahren kommen im Ayurveda bei zahlreichen chronischen internistischen und neurologischen Fragestellungen zum Einsatz.

Im Februar 2022 wurden von der WHO neue Benchmarks für Ausbildung und Praxis im Bereich Ayurveda veröffentlicht, die Phytotherapie spielt dabei eine wichtige Rolle [120]. Zur Behandlung von „Arthritiden“ werden häufig Kombinationspräparate auf der Basis z. B. von Myrrhe (*Commiphora mukul*) und Weihrauch (*Boswellia serrata*) oder auch Einzelpflanzen wie die Winterkirsche/Ashwagandha (*Withania somnifera*), Kurkuma (*Curcuma longa*) und Ingwer (*Zingiber officinale*) im Rahmen multimodaler Therapiekonzepte eingesetzt [77–79].

### 2. Überblick über die wissenschaftliche Evidenz in der Literatur

Die wissenschaftliche Evidenz für eine Wirksamkeit von Ayurveda ist schwach. Kontrollierte wissenschaftliche Langzeitdaten zum präventiven Effekt von Ayurveda liegen nicht vor.

Die komplexe individualisierte und multimodale Behandlung ist schwer durch (placebokontrollierte) prospektive Studien abzubilden. Wirkungen werden bislang im Wesentlichen durch dokumentierte individuelle Heilerfolge und eine seit über 2000 Jahren durchgehende Medizintradition in Südasien begründet.

Für Einzelsubstanzen wie Kurkuma oder Weihrauch liegen Hinweise aus experimentellen Untersuchungen zur Hemmung der Lipoxygenase vor [80–83]. Für Kurkuma gibt es auch zunehmend klinische Evidenz (Übersicht in [84]).

### 3. Wissenschaftliche Evidenz in der Rheumatologie

#### Arthrose.

In einer aktuellen Metaanalyse zur Anwendung von Weihrauchpräparaten bei Arthrose konnten nur 7 aus 513 infrage kommenden Publikationen berücksichtigt werden, in denen 545 Patienten eingeschlossen waren. Eine signifikante Besserung von WOMAC Schmerz, Steifigkeit und Funktion im Vergleich zu Placebo wurde berichtet. Die Zeitdauer bis zur Besserung betrug mindestens 4 Wochen [85].

Für Kurkuma als Monoextrakt liegt eine Evidenz aus einer hochwertigen randomisierten Studie mit 70 Patienten mit symptomatischer Gonarthrose und dokumentiertem günstigem Effekt auf Knieschmerzen und Gelenkfunktion vor [86].

Eine weitere methodisch hochwertige Studie vergleicht randomisiert und kontrolliert ein multimodales ayurvedisches Behandlungskonzept mit einem zuwendungsanalogen konventionellen Konzept am Beispiel der symptomatischen Behandlung der Gonarthrose. Diese Studie belegte eine signifikante Überlegenheit für den patientenzentrierten Endpunkt WOMAC während der Behandlung, aber auch einen bis zu einem Jahr anhaltenden Vorteil von Ayurveda [87].

Die aktuelle S2k-Leitlinie zur Gonarthrose erwähnt Phytotherapeutika, die in der ayurvedischen Medizin Verwendung finden [117]. Dazu gehören Weihrauchpräparate, von denen abgeraten wird. Zu Ingwer- und Kurkuma-Extrakt sei nach vorliegender Evidenz keine Aussage möglich. Diese Empfehlung spiegelt jedoch noch nicht die Komplexität der in der oben genannten Studie [87] eingesetzten multimodalen Behandlungen der ayurvedischen Medizin wider. Für die Einschätzung von Kurkuma lag die Studie von Henrotin et al. noch nicht vor [86].

#### Rheumatoide Arthritis (RA).

Die 2020 publizierte AWMF-Leitlinie zum Management der frühen rheumatoiden Arthritis [88] kann für eine Anwendung spezieller komplementärer Verfahren (… „indische Medizin“ …) aufgrund mangelnder Evidenz keine Empfehlung aussprechen. Sie sieht keine ausreichenden Belege aus kontrollierten Studien zu verschiedenen Rezepturen von nachvollziehbarer, höherer Qualität aus dem Gebiet des Ayurveda.

Zitiert wird hierzu nur eine systematische Übersichtsarbeit zur ayurvedischen Behandlung bei RA, die bereits 18 Jahre alt ist. Diese sieht keine ausreichende Evidenz für einen Nutzenbeleg [89].

Die individualisierte und konstitutionsgeleitete Therapiesteuerung der RA im Ayurveda wurde bislang nur in einer doppelblind randomisierten Studie untersucht. In dieser randomisierten Pilotstudie wurden in 3 Armen MTX plus Ayurveda-Placebo (*n* = 14), Ayurveda plus MTX-Placebo (*n* = 12) und Ayurveda plus MTX (*n* = 17) verglichen. Im Hauptergebnis waren die 3 Interventionen bezüglich DAS28 und ACR-Response nach 24 und 36 Monaten weitgehend äquivalent, ein signifikanter Unterschied konnte lediglich für MTX nach 24 Wochen bezüglich der ACR 70-Response dokumentiert werden. Die Ayurveda Therapie war insgesamt verträglicher. Einschränkend sind jedoch die sehr kleinen Kohorten, die nicht zum Beleg der Gleichwertigkeit genügen, und der hohe Anteil randomisierter, aber nicht ausgewerteter Patienten (*n* = 18). Die Behandlung im Ayurveda-Arm wurde individuell umgesetzt, damit bildet dieser Arm nicht die pharmakologische Wirkung einer standardisierten Substanz, sondern den Effekt von Vielstoffgemischen sowie die individuelle Statuserfassung des Patienten durch die Therapeuten ab. Dieses Konzept erlaubt keine Aussage zur generellen Wirkung einzelner Substanzen [90]. Eine analoge größere und langzeitigere Studie mit konfirmatorischem Studiendesign wird von der gleichen Arbeitsgruppe derzeit durchgeführt, Ergebnisse werden in 2023 erwartet.

RA‑1, eine an Ayurveda angelehnte Mischung aus 4 Substanzen – Schlafbeere (Ashwagandha) (*Withania somnifera*), Weihrauch (*Boswellia serrata*), Ingwer (*Zingiber officinale*) und Kurkuma (*Curcuma longa*) –, konnte in einer 16-wöchigen doppelblind randomisierten Studie keinen signifikanten Effekt erzielen [91].

In der folgenden Langzeitstudie mit RA‑1 bei 182 eingeschlossenen Patienten wurden 122 Patienten über 3 Jahre dokumentiert. Etwa die Hälfte der Patienten nahm RA‑1 ohne weitere DMARDs ein. Das Outcome war vergleichbar den Patienten mit DMARDs, allerdings zeigten Letztere bei freier Therapiewahl eine initial höhere Krankheitsaktivität. Patienten unter DMARDs hatten mehr Nebenwirkungen [92].

In einer 90-tägigen dreiarmigen Studie wurde Kurkuma (CuroWhite) in Dosierungen von 250 und 500 mg oder Placebo bei Patienten mit RA eingesetzt. Die Autoren beschreiben signifikante und deutliche Besserungen von DAS28 (um 50–64 %), BSG (um 88–89 %), CRP (um 31–45 %) und Rheumafaktor (um 80–84 %). Es wurden jeweils nur 8 Patienten je Arm eingeschlossen, davon nur die Hälfte weiblich. Die Studie ist in Statistik, Methodik und Ergebnis unglaubhaft und enthält schwerwiegende methodische Fehler [93].

In Deutschland wurden 2 randomisierte doppelblinde, placebokontrollierte Studien mit dem Weihrauchpräparat H15 bei ambulanten Patienten mit langjähriger RA durchgeführt, und es wurde eine signifikant dem Placebo überlegene Wirkung der Testsubstanz als Abstract ohne Studiendetails und Berechnungsgrundlagen veröffentlicht [94]. Eine Neuberechnung der Rohdaten konnte einen Vorteil von Weihrauch gegenüber Placebo bei der Add-on-Therapie der RA nicht belegen [95].

### 4. Mögliche Anwendungen in der Rheumatologie inklusive zu erwartender positiver Effekte

Aus den oben genannten Ausführungen ergibt sich, dass die Daten für die Therapie mit ayurvedischen phytotherapeutischen Einzelsubstanzen bei rheumatoider Arthritis keinen ausreichenden Effekt auf die etablierten Surrogatmarker für eine Kontrolle der Erkrankung zeigen. Für die individualisierte Therapie mit Vielstoffgemischen und multimodalen Behandlungen sind die Ergebnisse laufender Studien abzuwarten.

Für die Behandlung der Arthrose ergibt sich eine erste positive Evidenz für die ayurvedische multimodale Therapie sowie für die Verwendung von Kurkuma bzw. Curcumin-Extrakten als Einzelsubstanz. Die unzureichende Datenlage zu anderen rheumatischen Erkrankungen ermöglicht keine Beurteilung.

### 5. Mögliche Nebenwirkungen und Limitationen

Phytotherapien können verschiedene Neben- und Wechselwirkungen haben. Mögliche Belastungen könnten Toxine und Schwermetalle in pflanzlichen Extrakten unklarer Herkunft sein, v. a. wenn ungeprüfte Präparate aus unseriösen Quellen bezogen werden. Es besteht die prinzipielle Gefahr, dass eine notwendige DMARD-Therapie durch die Anwendung ayurvedischer Medizin verzögert begonnen wird.

### 6. Abschließende Empfehlung der Kommission

Die aktuelle Datenlage genügt nicht, die Empfehlung der Leitlinie zum Management der rheumatoiden Arthritis bezüglich Ayurveda zu revidieren, welche diese Methode bei RA nicht empfiehlt.

Die Umsetzung von Ayurveda über den Einsatz der Einzelsubstanzen hinaus bedarf einer qualifizierten Ausbildung, die in Deutschland über die Deutsche Ärztegesellschaft für Ayurveda-Medizin e. V. DÄGAM zertifiziert wird [121]. Wenn diese Qualifikation gegeben ist, kann bei degenerativen Gelenkerkrankungen die ayurvedische Medizin im Rahmen einer Komplexbehandlung zum Einsatz kommen.

Weitere Studien und Konzepte zur Überprüfung eines Zusatznutzens des ergänzenden Einsatzes von Ayurveda werden begrüßt.

## Empfehlungen der Kommission für Komplementäre Heilverfahren und Ernährung zur Anwendung der Homöopathie in der Rheumatologie

### 1. Definition der Methode [96]

Bedeutung des Begriffes: Homöopathie bedeutet „ähnliches Leiden“ (altgriechisch homóios, deutsch „gleichartig“, „ähnlich“, und páthos, deutsch „Leid“, „Schmerz“). Es handelt sich um eine Behandlungsmethode der Alternativmedizin, deren Theorie vom deutschen Arzt Samuel Hahnemann (geb. 1755 in Meißen; gest. 1843 in Paris) im 18. und 19. Jahrhundert entwickelt wurde.

Prinzipien der Homöopathie sind:

1. das Ähnlichkeits- oder Simileprinzip als wichtigste Grundannahme: „Ähnliches möge durch Ähnliches geheilt werden“ (lat. „similia similibus curentur“): Homöopathische Arzneimittel sollen an Gesunden ähnliche Symptome hervorrufen können wie die, an denen der Kranke leidet;
2. die homöopathische Arzneimittelprüfung an Gesunden, bei der homöopathische Arzneimittel eingenommen und die Symptome protokolliert werden, um Arzneimittelbilder zu erstellen;
3. die Erhebung des individuellen Krankheitsbildes durch homöopathische Anamnese. Bestandteile dieser Anamnese sind Spontanbericht, gelenkter Bericht, indirekte Befragung und biografische Anamnese;
4. das System der Potenzierung: Es beruht auf der Annahme von Hahnemann, dass durch eine Minimierung der Dosis eine Steigerung ihrer Wirksamkeit im Sinne einer Freisetzung verborgener dynamischer Kräfte erfolgen könne.

Die unter 1.) und 4.) genannten Prinzipien halten einer wissenschaftlichen Nachprüfung nicht stand.

### 2. Überblick über die wissenschaftliche Evidenz in der Literatur

Die Evidenz für eine Wirksamkeit der homöopathischen Therapie (HT) ist generell schwach [97], Wirkungen werden im Wesentlichen durch Placeboeffekte erklärt [98]. Cochrane-Analysen zur Wirkung bei respiratorischen Infekten im Kindesalter [99], Aufmerksamkeitsdefizitsyndrom (ADHS) [100], irritablem Kolon [101], Influenza und Influenza-ähnlichen Symptomen [102] und Demenz [103] fanden keine Effekte, zur HT bei Nebenwirkungen einer Chemotherapie wurden marginale Effekte für 2 Dermatika beschrieben [104]. Ein systematischer Review zur Anwendung der HT in der Dermatologie blieb negativ [105]. In einem der Homöopathie gewidmeten Journal wurde nach Auswertung von über 300 Veröffentlichungen zu homöopathischen Studien mangelhafte Studienqualität konstatiert, der Beweis für eine über Placeboeffekte hinausgehende Wirkung stünde noch aus [106]. Eine Sichtung aller zum Publikationszeitpunkt vorhandenen systematischen Reviews konnte ebenfalls keinen Wirkungsnachweis erbringen [21].

### 3. Wissenschaftliche Evidenz in der Rheumatologie

#### Rheumatoide Arthritis (RA).

Zwei frühe kontrollierte Studien mit gleichem Autor beschrieben an kleinen Fallzahlen positive Effekte der HT im Vergleich zu Placebo [107] oder Salizylaten [108]. Die Effektstärke ist von fraglicher Relevanz, die Methodik entspricht nicht heutigen Standards. Eine weitere kontrollierte Studie konnte nachweisen, dass der Effekt einer HT auf das intensive Anamnesegespräch, nicht jedoch auf die homöopathischen Medikamente zurückzuführen war [109]. Eine andere randomisierte, kontrollierte Studie blieb nach 3 Monaten ohne Effekt [110]. Zu erwähnen sind 2 Studien, bei denen entweder hochverdünnte Antikörper gegen TNF-alpha [111] oder hochverdünnte inflammatorische Zytokine [112] als potenzielle homöopathische Medikamente für die RA beschrieben werden, ohne dass dafür relevante klinische Daten vorliegen würden.

#### Spondyloarthropathien (SpA).

Eine deutschsprachige Publikation konnte in einer doppelblind randomisierten Studie keine Wirkung eines homöopathischen Präparates bei Patienten mit axialer SpA nachweisen [113].

#### Fibromyalgiesyndrom (FMS).

Eine Metaanalyse klinischer Studien zur Komplementärmedizin bei FMS konnte für die HT aufgrund schlechter Datenqualität keine Empfehlung abgeben [28]. Eine deutsche Leitlinienkommission für die Bewertung komplementärer und alternativer Heilverfahren für das FMS erzielte bei der Bewertung der HT keinen Konsens, daher wurde weder eine positive noch eine negative Empfehlung ausgesprochen [114].

#### Gelenkbezogene Schmerzen.

In einer placebokontrollierten Studie konnten homöopathische Mittel den postoperativen Analgetikaverbrauch nach Kniegelenkoperation nicht reduzieren [115].

#### Gonarthrose.

In der aktuellen Leitlinie zur Gonarthrose findet sich die Stellungnahme: „Zur Homöopathie bei Kniearthrose kann aufgrund der Studienlage keine Aussage gemacht werden“ [117].

#### Weitere rheumatische Erkrankungen.

Eine PubMed-Recherche ergab keine Veröffentlichungen unter den Stichworten „homeopathic“ oder „homeopathy“ und „Lupus“, „Scleroderma“ „Myositis“, „Giant Cell Arteriitis“, „Systemic Vasculitis“ und „Polymyalgia rheumatica“

### 4. Mögliche Anwendungen in der Rheumatologie inklusive zu erwartender positiver Effekte

Aus den oben genannten Ausführungen ergibt sich, dass für die HT bei entzündlichen Gelenkerkrankungen, autoimmunen Systemerkrankungen und Fibromyalgiesyndrom keine relevanten positiven Effekte zu erwarten sind.

### 5. Mögliche Nebenwirkungen und Limitationen

Homöopathische Medikamente gelten allgemein als gut verträglich, frei von unerwünschten Effekten sind sie jedoch nicht [116]. Ein nicht unwesentliches Problem bei Anwendung der HT könnte die Verzögerung einer suffizienten rheumatologischen Therapie sein [14].

### 6. Abschließende Empfehlung der Kommission

Die homöopathische Therapie wird für Patienten mit entzündlichen und degenerativen Gelenkerkrankungen, autoimmunen Systemerkrankungen und FMS nicht empfohlen.

## Schlussfolgerung

Die hier präsentierten Empfehlungen stellen eine Momentaufnahme dar, die dem aktuellen Stand wissenschaftlicher Veröffentlichungen entspricht. Für die Erstellung einer Leitlinie nach AWMF-Kriterien reichte die Qualität des Datenmaterials nicht aus, da qualitativ hochwertige Studien zur CAM in der Rheumatologie weitgehend fehlen. Die Kommission ist dringend daran interessiert, dieses Defizit in den nächsten Jahren zu verringern. Dennoch sollten die vorliegenden Ausarbeitungen als Ergebnis gründlicher Literaturrecherche und fachlicher Expertise der Kommissionsmitglieder eine brauchbare Hilfe für die Kommunikation mit PatientInnen in der täglichen Praxis sein.

Die Kommission plant in den kommenden Jahren die Ausarbeitung weiterer Empfehlungen zu den Themen „Traditionelle chinesische Medizin“ und „Phytotherapeutika“ sowie „Mind-Body-Medizin“, „Übergewicht“ und „Alkoholkonsum“.
